# Exploring Extremophiles from Bulgaria: Biodiversity, Biopolymer Synthesis, Functional Properties, Applications

**DOI:** 10.3390/polym16010069

**Published:** 2023-12-25

**Authors:** Songül Yaşar Yıldız, Nadja Radchenkova

**Affiliations:** 1 Department of Bioengineering, Istanbul Medeniyet University, 34720 Istanbul, Turkey; songul.yildiz@medeniyet.edu.tr; 2 The Stephan Angeloff Institute of Microbiology, Bulgarian Academy of Sciences, 1113 Sofia, Bulgaria

**Keywords:** extremophiles, thermophilic microorganisms, halophilic microorganisms, exopolysaccharides (EPSs), biopolymers

## Abstract

Bulgaria stands out as a country rich in diverse extreme environments, boasting a remarkable abundance of mineral hot waters, which positions it as the second-largest source of such natural resources in Europe. Notably, several thermal and coastal solar salterns within its territory serve as thriving habitats for thermophilic and halophilic microorganisms, which offer promising bioactive compounds, including exopolysaccharides (EPSs). Multiple thermophilic EPS producers were isolated, along with a selection from several saltern environments, revealing an impressive taxonomic and bacterial diversity. Four isolates from three different thermophilic species, *Geobacillus tepidamans* V264, *Aeribacillus pallidus* 418, *Brevibacillus thermoruber* 423, and *Brevibacillus thermoruber* 438, along with the halophilic strain *Chromohalobacter canadensis* 28, emerged as promising candidates for further exploration. Optimization of cultivation media and conditions was conducted for each EPS producer. Additionally, investigations into the influence of aeration and stirring in laboratory bioreactors provided valuable insights into growth dynamics and polymer synthesis. The synthesized biopolymers showed excellent emulsifying properties, emulsion stability, and synergistic interaction with other hydrocolloids. Demonstrated biological activities and functional properties pave the way for potential future applications in diverse fields, with particular emphasis on cosmetics and medicine. The remarkable versatility and efficacy of biopolymers offer opportunities for innovation and development in different industrial sectors.

## 1. Introduction

Life on Earth has shown remarkable adaptability, with organisms not just surviving but actively thriving in seemingly inhospitable environments that were once considered impossible for any biological activity. Over the years, scientific exploration, aided by advanced molecular tools, has revealed the existence of several microbiomes, such as microbial mats, stromatolites, and microbialites, possibly representing the descendants of the earliest inhabitants of our world [1,2]. These ancient microbial communities have endured and adapted to challenging environments, enduring conditions such as elevated temperatures close to the boiling point of water, frigid temperatures below the freezing point of water, intense radiation, low water availability, both acidic and alkaline pH levels, elevated salinity, extreme substrate concentrations, heavy metal contamination, and even the immense pressures of the deep ocean floor [3,4].

Although these extremophiles have inhabited some of the most challenging niches on the planet, they have captured the imagination of scientists and researchers alike due to their potential applications in various industries. Extremophiles have become valuable biological resources for their ability to produce unique compounds and biomolecules with exceptional properties. Their adaptive strategies, which include the biosynthesis of extremozymes capable of retaining their catalytic activity under harsh environmental stresses, have sparked great interest in understanding their biological processes and harnessing their potential for biotechnological advancements [3,5].

One particular group of biomolecules that has garnered significant attention is EPSs. These microbial exopolymers are high-molecular-weight polymers composed of sugar residues and represent a relatively new class of microbial products [6]. EPSs are not only intriguing for their structural complexity but also for their natural origin, non-toxicity, biocompatibility, and biodegradability. Moreover, the wide variety of properties exhibited by EPSs, stemming from the diversity in their composition, has further fueled the scientific fascination surrounding these compounds [7].

Industrially, EPSs have proven to be versatile substances, finding use as stabilizers, thickeners, gelling agents, coagulants, emulsifiers, lubricants, flocculants, and flavor enhancers across various sectors. Industries ranging from food production, brewing, cosmetics, and textiles to pharmacology, detergents, oil recovery, and wastewater treatment have all benefited from the applications of microbial EPSs [8]. While these biopolymers have been known since the 1880s, they have only gained significant industrial attention within the past three decades [9]. Among the already established industrial-scale EPS producers, compounds like xanthan and gellan have emerged as highly appreciated hydrocolloids on the global market [6,10].

In comparison to traditional plant polysaccharides, microbial EPSs present a host of advantages. The production of microbial EPSs is a much faster process, taking only a matter of days compared to the longer life cycles of plants, which can last for months or even years. Furthermore, microbial EPSs can be produced under controlled conditions, ensuring a consistent and reproducible yield, with the added benefit of the possibility to tailor the product’s composition to meet specific requirements [11].

One key distinction between bacterial and plant polysaccharides lies in their branching structures. Plant polysaccharides are mainly homopolysaccharides, while microbial EPSs generally lack significant branching and are predominantly heteropolysaccharides, often incorporating various chemical groups [12]. This chemical diversity results in a vast array of bacterial EPS structures, comprising three to seven different monosaccharides, frequently modified with acetate, pyruvate, succinate, sulfates, and phosphates. The incorporation of these substituents renders many EPSs polyanionic in nature [6]. Bonds between monomeric units at the backbones of the polymers are 1,4-β or 1,3-β linkages and 1,2-α or 1,6-α linkages. The former are characterized by strong rigidity, while the latter are more flexible ones [13]. In this sense, the type of binding relationships between monomer units in plant EPSs and bacterial ones is another differentiating factor. In general, the structure of polysaccharides is determined by the arrangement of the monosaccharide units, the type of glycosidic bonds between them, and the interactions between the various functional groups. These structural features contribute to the diverse functions and properties exhibited by polysaccharides in biological systems.

The investigation of the chemical characteristics of these carbohydrate polymers has not only provided insights into their environmental roles but has also unveiled their tremendous potential in diverse commercial applications [14,15]. Researchers have found 90 EPS producers in *Bacteria*, *Archaea*, and *Fungi* domains, with the majority being mesophilic microorganisms [3]. The isolation of nonpathogenic EPS producers holds promise for expanding their use in the food industry and opens new avenues for biotechnological processes in drug production, medical diagnosis, and the creation of new biodegradable plastics [16].

In recent years, the demand for new microbial polysaccharides with superior properties to existing polymers has steadily increased [17,18]. Researchers have sought to identify novel EPS producers, particularly from extremophiles, which are lesser-known microorganisms that have adapted to thrive in extreme habitats. Although the number of reports on EPS production by extremophiles is currently limited compared to mesophiles, these unique microorganisms have evolved exceptional metabolic and physiological capabilities to survive in extreme environments. Consequently, they offer a vast natural resource of commercially valuable products, including those produced via EPS biosynthesis.

Bulgaria, with its rich thermal springs of diverse geotectonic origins and varying mineral compositions, represents a promising source of thermophilic EPS producers with interesting biotechnological potential [19]. Scientists from the Laboratory of Extremophilic Bacteria at the Institute of Microbiology, the Bulgarian Academy of Sciences, have been actively involved in isolating potential thermophilic EPS producers with unique properties. Bulgaria is located in southeastern Europe and is a region rich in mineral springs. In the northern region of the country, cold water springs are the prevailing type, whereas in southern Bulgaria, warm springs with temperatures ranging from 37 °C to 60 °C, and hot springs exceeding 60 °C, are characteristic. These springs originate from diverse geotectonic sources, and they exhibit variations in both pH levels, with the latter falling within the range of 6.5 to 9.5, and temperatures. The warm and hot springs in southern Bulgaria are associated with nonactive volcanoes that possess hydrothermal activity, classifying them as part of the neutral to slightly alkaline category within the existing bimodal pH distribution of hot water [19]. The ecological niches of thermal springs situated in southeastern Europe have not received adequate research attention. On the other hand, within the territory of the country is located Atanasovsko Lake and the Pomorie salterns—there, the salinity of the water is 20–28, with rich plant, animal, and microbial diversity. Research on their biodiversity and the determination of phylogenetic affiliation enriches the knowledge in this direction. The production of EPSs stands as a strategic approach to facilitate the active proliferation of halophilic microorganisms. A good producer of extracellular polymeric substance was isolated: the moderately halophilic bacterium *Chromohalobacter canadensis* 28. The limited understanding of the overall diversity of thermophilic and halophilic microorganisms, coupled with a scarcity of research focused on extreme niches within Bulgaria, along with the potential for diverse applications of thermostable enzymes and EPSs, serves as a driving force for scientists to engage in these investigations.

The objective of this review is to offer an extensive examination of the synthesis, isolation, manufacturing, retrieval, and utilization of EPSs originating from extremophilic microorganisms, shedding light on their potential as valuable resources in the field of biotechnology. As extremophiles offer products that are nonpathogenic and suitable for use in the food, pharmaceuticals, and cosmetics industries, the study of EPSs produced by these microorganisms holds promise for various biotechnological applications. Furthermore, the review emphasizes the importance of exploring the biodiversity of extremophiles and isolating reliable EPS producers to unlock the full potential of these remarkable microorganisms in the realm of biopolymers and biotechnological innovations.

## 2. Thermophiles

### 2.1. Diversity in Bulgarian Hot Springs

Thermophilic microorganisms have been studied for a considerable period, yet they were often regarded as curious biological oddities. However, the burgeoning interest in biotechnology has spurred further research in this field, resulting in significant advancements in knowledge. One particularly promising avenue of study involves exploring biodiversity in thermal niches, which has led to the discovery of novel biologically active substances like enzymes and EPSs. These findings hold great ecological and environmental relevance, with potential applications in environmental protection [6,20].

To investigate this area, a comprehensive examination of archaeal and bacterial diversity was conducted in two distinct hot springs located in Bulgaria. These springs were located in different geographical areas, with differing tectonic origins and varying water temperatures. The study focused on the analysis of two genes, 16S rRNA and GH-57 (the glycoside hydrolase 57 family of proteins; the number of its members has grown and now includes more than 1100 bacterial and archaeal proteins) [21]. The results revealed that bacterial diversity was more abundant in the spring Vetren Dol (VD) (68 °C), whereas the hotter spring, Levunovo (LV) exhibited notably greater diversity among archaeal populations (82 °C).

Upon analyzing the LV library clones, a total of twenty-eight distinct sequence types were recognized, and classified into five distinct archaeal groups from *Euryarchaeota* and *Crenarchaeota*. The dominant groups were *Candidate*, *Thaumarchaeota*, and *Methanosarcinales*. Meanwhile, the majority of the clones from VD were classified under the HWCG (Hot Water Crenarchaeotic Group), suggesting the formation of a group of thermophiles within the order *Methanosarcinales*. Additionally, the phylogenetic analysis unveiled a considerable number of novel sequences, with more than one-third of the archaeal and half of the bacterial phylotypes showing less than 97% similarity to known ones [21].

In particular, the GH-57 gene analysis allowed for a higher resolution of biodiversity assessment and specific taxonomic group analysis. Moreover, the phylogenetic diversity of culturable bacteria from the *Bacillus* genus and closely related genera was investigated, which were isolated from 18 other hot springs across the country [22]. Within this group of strains, one strain was identified as belonging to the *Thermoactinomyces* genus, while the remaining sixty-six strains represented eight distinct species spanning four genera: *Brevibacillus*, *Geobacillus*, *Anoxybacillus*, and *Bacillus*. Upon conducting more in-depth phylogenetic analysis, it was determined that four of these strains fell into categories indicative of potentially novel species, as their 16S rRNA gene sequences exhibited less than 97% similarity to known sequences. Subsequently, these strains were formally characterized and described as new species, namely *Anoxybacillus rupiensis* [23] and *Anoxybacillus bogrovensis* [19].

Overall, these findings underscore the immense potential of thermophilic microorganisms as a valuable resource for biotechnological applications and contribute significantly to our understanding of microbial diversity in thermal environments, benefiting both scientific research and environmental protection efforts [24].

### 2.2. EPSs from Thermophilic Bacteria

In the past two decades, various microorganisms belonging to the Archaea and Bacteria domains, residing in extreme, hot environments such as volcanic sites, terrestrial geothermal springs, and deep-sea hydrothermal vents, have been extensively documented [25]. These microorganisms are categorized based on their optimal growth temperature: thermophiles, which thrive within the temperature range of 55 to 80 °C, and hyperthermophiles, which inhabit environments with temperatures exceeding 80 °C [26]. Within the realm of thermophiles, numerous EPS-producer microorganisms belonging to the *Bacilli* species, including genera such as *Aeribacillus*, *Anoxybacillus*, *Brevibacillus*, and *Geobacillus*, have been successfully obtained from geothermal springs in various countries, spanning Armenia, Antarctica, Bulgaria, China, Italy, and Turkey. Additionally, certain bacterial genera like *Rhodothermus* and *Thermogata*, as well as archaeal genera like *Sulfolobus* and *Thermococcus*, have also demonstrated their capacity to produce EPS [27,28].

Thermophilic organisms exhibit diverse metabolic capabilities, utilizing a range of sugars like lactose, glucose, and sucrose as carbon sources, and relying on amino acids or complex sources such as organic nitrogen sources for both growth and EPS production. The composition of the sugar medium significantly influences the yield and chemical composition of EPS [29]. For instance, research has shown that different thermophilic strains, which were isolated from marine hot springs and shallow hydrothermal vents, can make use of a variety of carbohydrates [30]. Interestingly, certain strains have exhibited significant enhancements in EPS production, reaching up to a thousandfold increase, when they are supplied with particular sugars such as trehalose [31]. The choice of nitrogen source also influences EPS production. For instance, the archaeon *T. litoralis* is capable of thriving and producing EPS in a defined medium using amino acids as the exclusive nitrogen source, regardless of whether maltose is present or absent. Conversely, *T. maritima* can only grow in a medium supplemented with maltose and NH_4_Cl and cannot utilize amino acids as sources of carbon, nitrogen, or energy [29].

Aside from their remarkable EPS-producing capabilities, thermophilic organisms typically exhibit high growth rates and achieve maximum EPS production in shorter fermentation periods compared to mesophilic organisms. As temperature increases, the solubility of oxygen in the medium decreases, impacting the production of enzymes involved in EPS metabolic pathways [32]. Consequently, the yields are often relatively modest. However, cultivating EPS-producing thermophiles and hyperthermophiles offers several advantages, including shortened fermentation processes, faster growth rates, reduced susceptibility to contamination by mesophiles, and decreased broth viscosity as temperature rises, enhancing mass transfer when proper aeration and agitation are provided. These advantages have spurred the development of various strategies for upscaling the fermentation process [7,16].

Continuous culture methods have also demonstrated significant advantages for thermophilic microorganisms, as they maintain cultures in the exponential growth phase due to their rapid growth rates. For instance, thermophilic *Aeribacillus pallidus* 418 achieved nearly twofold higher yields of biomass and EPS in continuous culture compared to batch cultivation [33,34,35]. Furthermore, the chemical composition of EPS is notably affected by the cultivation mode, with continuous culture resulting in increased carbohydrate content and decreased protein content when compared to batch culture.

The synthesis of exopolysaccharides is believed to be an adaptation mechanism for the survival of microorganisms in harsh conditions. However, the relationship of thermophilicity to monosaccharide composition and structure has not been sufficiently studied. The optimized carbon sources for most thermophilic bacteria for EPS production are disaccharides such as maltose, sucrose, and lactose. The type of sugar subunits and other constituents plays a pivotal role in determining their thermal stability. For instance, the strong inherent interactions among monosaccharides in EPS1-T14 and EPS-B3-15, produced by two strains of *Bacillus licheniformis*, have been attributed to their high thermal stability [36,37] Thermophilic EPSs also exhibit exceptional thermostability, with some EPSs undergoing thermal degradation at high temperatures, while others remain stable even at elevated temperatures. Corsaro et al. [38] suggest that species specificity of the EPS is produced. They assume that bacteria originating from the same species produce EPSs, containing the same type of monosaccharides. For example, exopolysaccharides isolated from two strains of *Geobacillus* sp. contain glucose as the main sugar and mannose and galactose in different proportions [39], and a third strain from the same genus contains glucosamine and arabinose together with galactose and mannose. Further research into the influence of monosaccharide molar ratios on EPS thermostability is warranted. Numerous studies have reported on the biological activities of EPSs produced by thermophilic organisms, indicating their potential applications across various fields [40,41,42,43] (Table 1).

### 2.3. Geobacillus tepidamans V264

*Geobacillus tepidamans* V264, first isolated from the Mizinka hot spring situated in Velingrad, Bulgaria [44], demonstrated noteworthy characteristics in its EPS production. Maltose and (NH_4_)_2_HPO_4_ were identified as the most suitable carbon and nitrogen sources, respectively, at levels of 30 and 3 g/L. The use of an inorganic nitrogen source by this isolate for EPS production shows significant potential for designing cost-effective industrial media.

In contrast to mesophilic processes that can span several days, *Geobacillus tepidamans* V264 achieved peak EPS production within just 8 h of cultivation in a bioreactor operating at an optimal temperature of 60 °C and pH of 7.0. Monosaccharide analysis of the EPS indicated that it primarily consisted of α-glucan, comprising more than 98% glucose, along with minor quantities of galactose, fucose, and fructose. Notably, glucose also dominated the polymer produced by *Thermotoga maritime*, exceeding 90% of the composition, while additional sugars such as ribose and mannose were present [45]. EPSs isolated from various *Geobacillus* sp. strains exhibited varying proportions of glucose, galactose, and mannose, and some strains included glucosamine and arabinose along with galactose and mannose in their EPS composition [39].

The demonstrated significant EPS production capabilities of *G. tepidamans* V264 present a compelling case for its utilization in various industrial sectors. The polymer’s exceptional molecular weight, reaching some of the highest reported levels for microbial EPSs, opens up possibilities for applications where substantial viscosity is a critical factor. This could be particularly advantageous in industries requiring thickening agents, such as in the formulation of paints and adhesives, or even in the food industry for improving texture and mouthfeel [46,47].

Moreover, the observed resistance of the EPS to decomposition at high temperatures not only enhances its appeal for industrial processes but also suggests potential applications in extreme environments. Industries requiring materials with structural stability under harsh conditions, such as those in aerospace or oil and gas, might find this polymer beneficial [48]. Moreover, the outstanding structural stability of the EPS, demonstrated by its ability to withstand decomposition at remarkably high temperatures (280 °C), enhances its benefits in handling and storage. This quality holds particular significance in the food, cosmetic, and pharmaceutical industries. This level of thermostability is comparable to other reported biopolymers, such as *G. thermodenitrifacans* B3-72 (240 °C) [31], as well as EPS1 and EPS2 from *Geobacillus* sp. strain WSUCF1, with reported stabilities of 319 °C and 314 °C, respectively [49].

The prevalence of glucose in the polymer’s composition adds an eco-friendly dimension to its potential applications [50]. With the increasing emphasis on sustainable practices, the EPS from *G. tepidamans* V264 could serve as a greener alternative to traditional thickeners derived from non-renewable resources. In terms of pharmaceutical applications, the neutral impact of polysaccharides on the metabolic processes of living organisms is a significant advantage. This property opens up possibilities for the development of a new class of anti-cytotoxic drugs with potentially fewer side effects compared to existing agents.

Looking forward, further exploration of the EPS’s functional properties and its interaction with different substrates can unlock even more diverse applications. The compatibility of *G. tepidamans* V264’s EPS with various industries, including pharmaceuticals, paints, and food, should be thoroughly investigated. Additionally, optimizing fermentation conditions to maximize EPS yield and tailoring its properties for specific applications can further enhance its versatility.

The study lays the groundwork not only for immediate applications but also for ongoing research that can lead to innovative uses of extremophilic microorganisms in biotechnology. Harnessing the unique properties of *G. tepidamans* V264’s EPS has the potential not only to address current industrial needs but also to inspire new solutions for challenges in various sectors.

### 2.4. Aeribacillus pallidus 418

*Aeribacillus pallidus* 418, isolated from Bulgaria’s Rupi basin, has been the focus of research into EPS synthesis [33]. High yields and economic efficiency are crucial factors in production processes. It is widely recognized that polymer synthesis is influenced by various factors, including the microorganism type, culture media, and engineering parameters such as vessel geometry, stirring device type, and oxygen transfer [51]. The scarcity of studies addressing the synthesis of extracellular biopolymers by thermophilic microorganisms has directed scientific attention toward this crucial aspect. The study sought to optimize conditions for EPS production and achieved a maximum yield of 170 mg/L with maltose and NH_4_CL as the best carbon and nitrogen sources, at 55 °C and pH 7.0. This impressive result was obtained in a single impeller monoagitator system known as Narcissus, operating at an agitation speed of 900 rpm and an aeration rate of 0.5 vvm with maximum productivity at the eighteenth hour. Notably, the research found that the agitation speed had a more substantial impact on EPS production than the aeration rate [34].

In-depth analysis led to the determination of two crucial oxygen transfer parameters: the oxygen uptake rate and the oxygen mass transfer coefficient (K_Lα_). These parameters played a pivotal role in understanding the bioprocess and its potential for scale-up, allowing a smooth transition from laboratory bioreactors to semi-industrial and industrial fermenters. Interestingly, the oxygen uptake rate was most pronounced under conditions mirroring the highest biomass viability, namely at 900 rpm and 0.5 vvm. In contrast, K_Lα_ exhibited a strong dependency on culture viability, increasing with higher growth rates, mixing intensity, and aeration rate [34].

Continuous cultivation experiments, particularly at a low dilution rate (D = 0.06), revealed a slight increase in EPS production. Moreover, continuous cultivation exhibited the advantage of producing biopolymers with a higher degree of purity, ranging from 95.3% to 97%, compared to batch cultures, which achieved only 80.5% purity [35]. The engineering aspects of studies under batch mode and continuous cultivation make it possible to assess the benefits of working with thermophilic producers. On the one hand, the possibility of contamination is reduced, and on the other, in the concrete work, the purity of the polymer fraction is increased.

Two distinct heteropolysaccharides were acquired following the purification of the polymer fraction. These were identified as electroneutral EPS 1 and negatively charged EPS 2. They were present in a relative weight ratio of 3:2.2 and exhibited an unusually diverse range of sugars, with EPS 1 containing six different types and EPS 2 containing seven [33]. The primary sugar component in EPS 1 was mannose (comprising 69.3% of its composition). Additionally, smaller amounts of glucose (11.2%), galactosamine (6.3%), glucosamine (5.4%), galactose (4.7%), and ribose (2.9%) were identified in EPS 1. Similarly, EPS 2 was primarily composed of mannose (33.9%), followed by galactose (17.9%), glucose (15.5%), galactosamine (11.7%), glucosamine (8.1%), ribose (5.3%), and arabinose (4.9%). *A. pallidus* YM-1, isolated from China [52], is another noteworthy representative of the same species. In contrast to *A. pallidus* 418, when glucose is included as the main carbon source, this strain synthesizes a biopolymer with the following monosaccharide composition: glucose (36.6%), altrose (30.9%), mannose (24.4%), galactose (8.1%), and a molecular weight of 526,369 Da. Isolated EPS has good emulsifying properties and promising potential in biotechnology. As expected, different nutrient media and culture conditions lead to variations in the composition of the EPS and, consequently, different physicochemical properties. Harichi et al. [53] documented the characteristics of the polyextremophilic strain *A. pallidus* CCUG 72355, demonstrating its proficiency in reducing concentrations of manganese, copper, aluminum, and nickel in treated vinasse. The EPS produced by this strain exhibits the capability to capture suspended solids, leading to a reduction in heavy metal content within the vinasse. This strain showcases significant potential for application in the remediation of polluted areas, contributing to ecological improvements overall.

**Figure 1:**
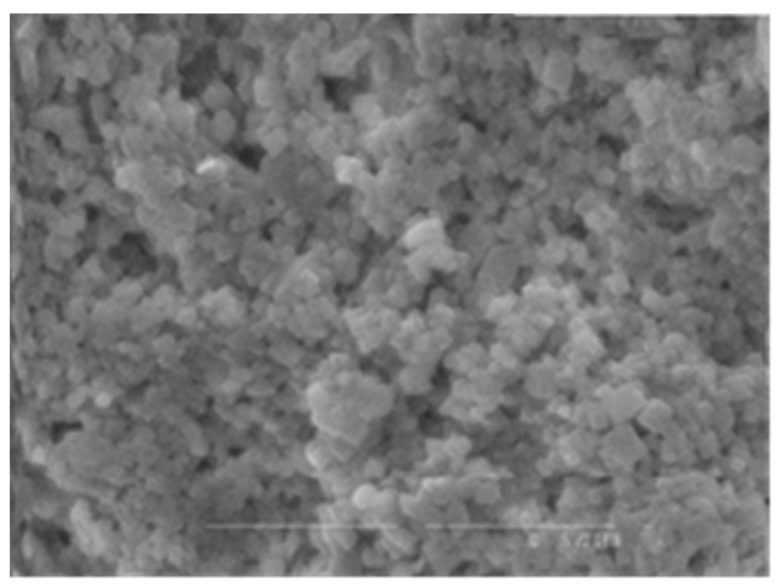
Microphotography of EPS 1.

**Figure 2:**
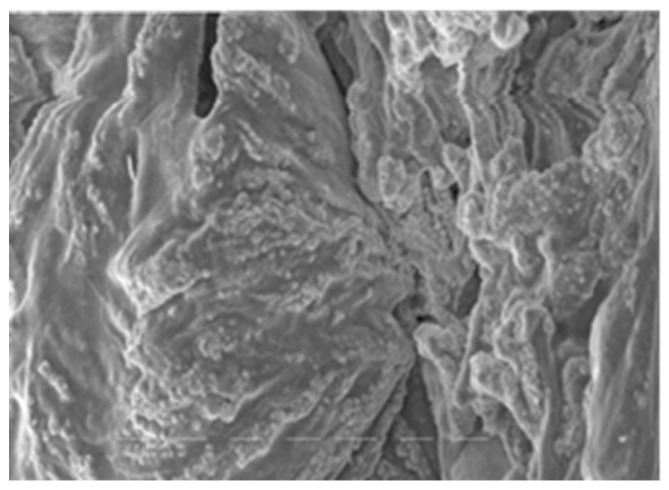
Microphotography of EPS 2.

Both of the polymers from *A. pallidus* 418 exhibited exceptional thermostability and boasted molecular masses that ranked among the highest reported for thermophilic producers, with EPS 1 reaching 700 kDa and EPS 2 exceeding 1000 kDa. Consequently, these EPSs have the potential to confer substantial viscosity to industrial products while requiring relatively small quantities of polymer. It is worth noting that EPSs from thermophilic bacilli have also been reported to possess molecular masses exceeding 300 kDa [33]. Both EPS 1 (Figure 1) and EPS 2 (Figure 2) were characterized by their anionic nature, attributed to the presence of uronic acids within their structures. This property is particularly advantageous in cosmetics due to the exceptional hydrating capabilities of uronic acids.

Figure 1 and Figure 2 show the structures of synthesized exopolymers from *A. pallidus* after lyophilization, observed with scanning electron microscopy [54].

The synthesized EPSs exhibited an exceptional ability to generate foam. Even at low concentrations, they produced significant foam, reaching up to 100% in volume compared to the water solution with a 1.5% EPS content. This attribute is especially valuable for applications requiring foaming [35]. Significantly lower foam stability, 22%, was reported for an exopolymeric substance synthesized by the filamentous cyanobacterium identified as *Leptolyngbya* sp. IkmLPT16 [55]. Moreover, incorporating EPSs by *A. pallidus* 418 into a basic cream led to notable changes. The cream’s viscosity increased at low shear rates, and it displayed pseudoplastic behavior. Furthermore, the yield stress of the cream, representing the maximum value in the shear stress–time relationship at a constant shear rate of 1 s^−1^, notably increased. This suggested the formation of a stable polymer network within the cream. Interestingly, the mechanical hysteresis for the cream containing 1% or 2% EPS remained constant, indicating that 1% EPS was sufficient to create a stable network, and the network’s strength did not significantly depend on EPS concentration [35].

The results of this study unveil the unique capability of *A. pallidus* 418 in producing exopolysaccharides with distinct characteristics, including diverse sugar compositions, impressive molecular weights, thermal stability, anionic properties, and exceptional foaming attributes. These features highlight the versatility of these EPSs across a broad spectrum of industrial applications, ranging from cosmetic products to those requiring high viscosity and foaming capabilities.

Furthermore, the study demonstrates that EPSs obtained from *A. pallidus* 418 can be produced at higher purity under continuous culture conditions, emphasizing their existence as heteropolysaccharides. These heteropolysaccharides are identified as electroneutral EPS 1 and negatively charged EPS 2. The unique sugar compositions, thermal stability, and the high reported molecular weights for microbial EPS of EPS 1 and EPS 2 suggest a significant potential for imparting substantial viscosity to industrial products.

In the future, further research can be conducted on the application of EPSs derived from *A. pallidus* 418, particularly in the cosmetic industry and applications requiring foaming. Additionally, additional research and development efforts may be necessary to optimize the performance of these EPSs in industrial products and tailor them for specific applications. This study lays a valuable foundation for the future exploration of the production and properties of thermophilic bacterial EPSs, contributing to potential advancements in biotechnological applications.

### 2.5. Brevibacillus thermoruber 423

*Brevibacillus thermoruber* 423 was isolated from the Gradechnitsa hot spring in the Blagoevgrad region of southwest Bulgaria. The highest EPS production by *B. thermoruber* 423 was achieved at 55 °C and pH 6.5 in the presence of maltose (18 g/L) and peptone (1 g/L). This carbon-to-nitrogen source ratio is commonly encountered in EPS production processes. The highest EPS production (897 mg/L) was observed at the early stationary phase in a bioreactor, and the yield and productivity were comparable to those of mesophilic organisms [56].

The results of chemical characterization indicated that the EPS obtained from *B. thermoruber* 423 was a heteropolymer composed of specific proportions of glucose, galactose, mannose, galactosamine, and mannosamine. Potential genes associated with EPS production were identified through genome analysis of the organism, and a hypothetical model for EPS biosynthesis was developed.

This study represented the initial comprehensive examination of EPS generated by *B. thermoruber* 423. Furthermore, this strain outperformed other thermophilic producers due to its impressive EPS synthesis capability. The fundamental proposition in this investigation was that *B. thermoruber* 423 will leverage its nonpathogenic attributes and rapid thermophilic productivity, positioning it as an exceptionally promising prospect for microbial EPS production. The research primarily focused on uncovering the intricacies of polysaccharide production in *B. thermoruber* strain 423 and identifying opportunities for potential enhancements [56]. This study aimed to identify high-level EPS producers as model organisms for the study of biological mechanisms related to EPS production. *B. thermoruber* 423 exhibited growth-associated EPS synthesis, shorter production times compared to mesophilic organisms, and high EPS yield and productivity. Additionally, the EPS produced displayed low viscosity and was sensitive to Ca^2+^ ion concentration. These characteristics make it a potential candidate for various industrial applications, including biomedicine [56].

Furthermore, the genome analysis of *B. thermoruber* 423 provided insights into the genetic and metabolic organization of the organism, paving the way for genetic and metabolic optimization for future biotechnological applications. This bacterium not only stands out as one of the few known thermophilic producers of EPS, but it also surpasses other thermophilic producers in terms of its remarkable polymer synthesis capabilities. Employing a systems-based approach, a comprehensive whole-genome analysis of this bacterium was conducted to gain deeper insights into the biological mechanisms and overall genomic organization of thermophilic EPS producers. This endeavor aimed to formulate rational strategies for enhancing EPS production through genetic and metabolic optimization. Additionally, this study represents the first instance of genome analysis conducted on a thermophilic *Brevibacillus* species. Through genome annotation, essential genes associated with EPS biosynthesis were identified, and in conjunction with experimental evidence, a hypothetical mechanism for EPS production was proposed. The study exhibited significant potential of *B. thermoruber* 423 in various biotechnological and industrial applications due to its ability to utilize xylose and produce EPS, isoprenoids, ethanol/butanol, lipases, proteases, cellulase, and glucoamylase enzymes, and for its resistance to arsenic [57].

To expedite progress in biotechnology and industrial applications, this study introduces a genome-scale metabolic model (GSMM) for *B. thermoruber* 423. Utilizing the recently acquired high-quality genome sequence, we constructed and subsequently validated this model by analyzing physiological data linked to batch growth and EPS production using seven distinct carbon sources. The developed model encompasses 1454 reactions, of which 1127 are associated with an enzyme commission number, and includes 1410 metabolites derived from 925 genes. This GSMM has the potential to streamline and accelerate further research in systems biology and large-scale industrial investigations, offering the capability to compute metabolic flux distribution within intricate networks and integrate data from various omics sources [58].

Exopolysaccharides produced by microorganisms like *B. thermoruber* 423 exhibit immense potential for diverse applications, laying the groundwork for future studies and innovations. The unique properties of EPS, including its composition, molecular weight, and functional characteristics, open up a wide range of possibilities for various fields. One promising avenue for the application of microbial EPS lies in the biomedical industry. The ability of *B. thermoruber* 423 to produce EPS with low viscosity and sensitivity to Ca^2+^ ion concentrations suggests its potential use in medical formulations, where controlled viscosity and ion sensitivity are critical factors [59,60]. The EPS, with its non-cytotoxic nature, could be explored for drug delivery systems, wound healing applications, and as a component in tissue engineering. Moreover, the resistance of *B. thermoruber* 423 to arsenic adds another dimension to its potential applications. The unique combination of EPS production and arsenic resistance suggests its potential role in bioremediation, especially in environments contaminated with arsenic [61,62]. Future studies could delve into optimizing conditions for EPS production in arsenic-rich environments and explore the use of *B. thermoruber* 423 in bioremediation processes. In the realm of bioprocessing, the EPS synthesized by *B. thermoruber* 423 could find applications as a stabilizing agent or thickener in various industrial products. Its ability to produce valuable products such as isoprenoids, ethanol/butanol, lipases, proteases, cellulase, and glucoamylase enzymes further enhances its versatility in biotechnological processes.

Looking ahead, future research could focus on genetic and metabolic engineering to enhance the production yield and optimize the properties of EPSs. Understanding the biosynthetic pathways and regulatory mechanisms involved in EPS production by *B. thermoruber* 423 could provide insights for targeted modifications, tailoring EPSs for specific applications. Additionally, exploring the interactions of *B. thermoruber* 423’s EPS with different substrates and its compatibility with diverse industries could uncover novel applications. As advancements in synthetic biology and genetic manipulation continue, the potential for creating designer EPSs with tailored properties becomes an exciting avenue for exploration [63].

In summary, the study of *B. thermoruber* 423’s EPS production not only reveals its immediate applications but also sparks enthusiasm for future investigations and innovations. The versatility of EPSs in biomedical, bioremediation, and industrial applications, coupled with the potential for genetic optimization, positions *B. thermoruber* 423 as a valuable asset in the landscape of microbial EPS research.

### 2.6. Brevibacillus thermoruber 438

*Brevibacillus thermoruber* 438 was isolated from a hot spring in the Rupi region in southwest Bulgaria [64]. This study explored the potential of *B thermoruber* 438 as an efficient producer of EPS. The strain exhibited affinity for various sugars as carbon sources, with maltose showing particular promise and (NH_4_)_2_HPO_4_ as the best nitrogen source. The research involved optimizing fermentation parameters to enhance EPS yield. EPS production initiated during the exponential growth phase and reached its peak after eight hours. These short fermentation processes are economically efficient. Optimal conditions for EPS production were found at a temperature of 55 °C and pH 8.0. Due to the significant impact of physicochemical factors on thermophilic growth and EPS production, considerable effort was dedicated to refining the fermentation procedures. During the exploration of the ideal temperature for polymer synthesis, the greatest amount of EPS (78.1 mg/L) was achieved at 55 °C. Elevating the temperature by just 5 °C led to a 35% reduction in EPS production, resulting in 50.53 mg/L. *B thermoruber* 438 exhibited substantial potential as an efficient producer of EPS in a brief eight-hour fermentation process. By fine-tuning culture conditions, polymer synthesis was nearly tripled, offering an exciting avenue for enhanced EPS production [64]. Looking forward, future studies could delve into further optimizing culture conditions to enhance polymer synthesis, aiming for even higher EPS yields. Exploring the genetic and metabolic underpinnings of EPS production in *B. thermoruber* 438 may provide insights for targeted modifications, potentially elevating its efficiency as an EPS producer. Additionally, investigating the applications of the synthesized EPS in various industries, such as biomedicine or bioprocessing, could unveil novel uses and contribute to the growing field of microbial EPS research. In essence, *B. thermoruber* 438’s demonstrated potential opens the door for exciting advancements in EPS production and utilization in the future.

**Table 1:** Thermophilic bacilli, carbon source, monosaccharide analysis, properties and activities, EPS production levels.

Thermophilic Bacilli (Specific Conditions)	Carbon Source	Monosaccharide Analysis	Properties and Activities	EPS Yield (g/L)	References
*Bacillus sonoresis* NTV10 (45 °C, pH 7.0)	Glycerol	Monosaccharide analysis: glucose/mannose/rhamnose in a relative ratio of 5.1:2.2:1	Food processing, cosmetics, and pharmaceuticals	1.597	[65]
*Bacillus haynesii* CamB6 (55 °C, pH 5.8)	Glucose	Monosaccharide analysis: mannose/glucose/galactose (3.3/1.0/0.7 by relative ratio)	Antioxidant, flocculation capacities, additive for the food industry	5.6	[66]
*Brevibacillus borstelensis* (50 °C, pH 6.4)	Glucose	Monosaccharide analysis: glucose and galacturonic acid	Bioemulsifier, stabilizer, bisurfactant, viscosifier and binding agent	1.88 ± 0.02	[67,68]
*Geobacillus* sp. *strain* WSUCF1 (60 °C, pH 7.0)	Glucose	EPS 1, Monosaccharide analysis: α-(1,3)-D-mannose and α-(1,6)-D-glucose (1/0.21, by molar ratio) EPS-2, Monosaccharide analysis: α-(1,3)-D-mannose	Both showed antioxidant activities, non-cytotoxicity	0.525	[49]
*Geobacillus thermodenitri-ficans* ArzA-6 (65 °C, pH 7.0)	Fructose	Monosaccharide analysis: mannose/galactose/arabinose/fructose/glucose (1/0.13/0.1/0.06/0.05, by relative ratio)	Not tested	0.27	[69]
*Geobacillus toebii* ArzA-8 (65 °C, pH 7.0)	Fructose	Monosaccharide analysis: mannose/galactose/glucose/arabinose (1/0.5/0.2/0.05, by relative ratio)	Not tested	0.22	[69]
*Geobacillus* sp. *TS3-9* (55 °C, pH 8.0)	Lactose	EPS 1, Monosaccharide analysis: mannose/trehalose/galactosamine/glucosamine/galactose/glucose/ribose (69.3/7.8/6.3/5.4/4.7/3.4/2.9, by molar ratio) EPS 2, Monosaccharide analysis: mannose/galactose/glucose/galactosamine/glucosamine/ribose/arabinose (33.9/17.9/15.5/11.7/8.1/5.3/4.9, by molar ratio)	Antioxidant activity, antitumor activity	0.087	[70]
*Geobacillus tepidamans* V264 (60 °C, pH 7.0)	Maltose	Monosaccharide analysis: glucose/galactose/fucose/fructose (1/0.07/0.04/0.02, by molar ratio)	Degradation temperature 280 °C, anti-cytotoxicity	0.111	[44]
*Aeribacillus pallidus* 418 (55 °C, pH 7.0)	Maltose	EPS 1, Monosaccharide analysis: mannose/trehalose/galactosamine/glucosamine/galactose/glucose/ribose (69.3/7.8/6.3/5.4/4.7/3.4/2.9, by molar ratio) EPS 2, Monosaccharide analysis: mannose/galactose/glucose/galactosamine/glucosamine/ribose/arabinose (33.9/17.9/15.5/11.7/8.1/5.3/4.9, by molar ratio)	Degradation temperature EPS1 176 °C, EPS2 226 °C, pseudoplastic rheological property, foaming ability, emulsifying activity	0.17	[33,34,35]
*Brevibacillus thermoruber* 423 (55 °C, pH 6.5)	Maltose	Monosaccharide analysis: glucose/galactose/mannose/galactosamine/mannosamine (57.7/16.3/9.2/14.2/2.4, by percentage of abundance)	Biocompatibility, non-cytotoxicity, stabilizing agents or thickeners	0.897	[56]
*Brevibacillus thermoruber* 438 (55 °C, pH 8.0)	Maltose	-	-	0.078	[64]

## 3. Halophiles

### 3.1. Diversity in Bulgarian Salt Habitats

Extremophiles, microorganisms capable of thriving in extreme environmental conditions such as extreme temperatures, high salinity, limited water availability, and extreme pH levels, are distributed worldwide across all three domains of life: Archaea, Bacteria, and Eukarya. Within this diverse group, halophiles are particularly noteworthy due to their adaptation to a wide spectrum of salt concentrations. Extreme saline environments, in particular, serve as unique ecosystems harboring untapped microbial biodiversity, making them a subject of great interest.

One specific study conducted by Kambourova et al. in 2017 investigated the bacterial and archaeal communities inhabiting P18, the largest salt lake in the Pomorie saltworks, characterized by an extreme salinity level of 34% [71]. The findings of this study diverged from previous reports of low bacterial diversity in hypersaline environments and instead revealed an exceptionally high diversity of taxa. Notably, several genera not previously identified as dominant in such environments were found to dominate the community. Moreover, this research led to the discovery of previously unknown 16S rRNA sequences.

Within the bacterial community, 23 bacterial operational taxonomic units (OTUs) were identified, affiliated with 15 bacterial genera spanning four phyla: Firmicutes (47.5%), Proteobacteria (23.1%), Bacteroidetes (22%), Deinococcus–Thermus (2.4%), and the candidate division SR1 (4.8%). The phylum Firmicutes was particularly prevalent, representing nearly half of the retrieved sequences.

In the archaeal domain, 26 distinct OTUs were clustered into 15 different genera from two orders, Halobacteriales and Haloferacales. These sequences were closely related to known halophiles, both culturable and unculturable, primarily from saline (often hypersaline) environments. Impressively, more than half of the archaeal OTUs corresponded to new sequences, with some forming distinct branches sharing 90% sequence similarity with their nearest relatives. These findings significantly deviated from prior investigations in terms of the number of genera identified, the dominance of certain genera not previously associated with such extreme niches, and the revelation of previously undiscovered 16S rRNA sequences.

Briefly, this research unveiled the existence of halophilic bacterial genera that had not previously been associated with other saltworks. The remarkable bacterial diversity observed in this hypersaline niche, characterized by limited similarity to known culturable representatives and low resemblance to unculturable clones, stands as a distinctive feature. This phenomenon can be attributed to the relatively limited understanding of halophilic bacteria in hypersaline environments. Moreover, the substantial proportion of newly identified bacterial and archaeal sequences indicates the potential presence of novel taxa across various taxonomic levels within the studied environment.

### 3.2. EPSs from Halophilic Bacteria

In the realm of halophilic microorganisms, many display the capacity to synthesize EPSs. These biomolecules likely serve to shield microbial cells from environmental stressors, particularly in the presence of high salt concentrations. They fulfill this role by regulating osmotic pressure and, effectively, alleviating the physical stress associated with salinity. This protection aids in maintaining membrane integrity [72,73]. The most common monosaccharide moieties in halophilic EPSs are mannose and glucose, some of which usually contain significant amounts of uronic acids and sulfates. Salinity changes affect biosynthesis, especially the ratio for the type of monosaccharide composition. It is acknowledged that to protect the microorganism from increasing salinity, the monosaccharide components in EPS may need to be modified in order to maintain its functions. For example, in biopolymer from *Aphanothece halophytica* GR02, when the NaCl concentration in the medium was elevated from 0.5 to 2.0 M, the proportions of galactose and rhamnose decreased, in contrast with the proportions of glucose and arabinose when the concentration of NaCl increased. Meanwhile, the monosaccharides in the EPS at different salinities stayed the same [74]. This indicates that the increase in glucose and arabinose and the decrease in galactose and rhamnose in the EPS secreted by *Aphanothece halophytica* GR02 may be advantageous to its survival in a high-salinity environment. Despite the accumulation of results on the monosaccharide composition and structure of exopolysaccharides, it still remains a challenge for scientists to determine the influence of monosaccharide molar ratios on the persistence of EPS at high salt concentrations.

In the *Bacteria* domain, numerous halophilic organisms are proficient EPS producers. For instance, moderately halophilic *Halomonas* spp. possess this capability. *H. maura*, notably isolated from a saline soil near a solar saltern in Asilah, Morocco, produces an anionic sulfated heteropolysaccharide known as mauran, characterized by a high uronic acid content and valuable functional properties [75]. *H. stenophila* strain HK30 and *H. rifensis* strain HK31^T^, both isolated from a soil sample near a solar saltern in Morocco, secrete EPS. The exopolymer from *H. stenophila*, named haloglycan, has a specific composition, including carbohydrates, acetyls, sulfates, sodium, uronic acids, phosphorus, and calcium [76,77] (Table 2). Strains *of H. eurihalina*, isolated from saline soils, also produce EPS, with strain H212 yielding the highest amount at 1.6 g/L [78]. *H. smyrnensis* AAD6^T^, isolated from the Çamaltı Saltern area in Turkey, is capable of producing high levels of levan using various carbon sources [79,80,81]. *Halomonas alkaliantarctica* strain CRSS, isolated from salt sediments near Cape Russell in Antarctica, exhibits the capacity to produce different EPSs under varying growth conditions, including mannan and xylo-mannan on complex media and fructo-glucan on minimal medium [14,82]. Meanwhile, *H. nitroreducens* strain WB1, found in a hydrothermal vent, produces an anionic EPS during the log and early stationary phases when grown on malt extract/yeast extract medium, primarily composed of glucose (Glc), mannose (Man), and galactose (Gal) [83]. *H. cerina* SP4^T^, isolated from saline soil in Spain, is a moderately halophilic microorganism known for EPS production [84].

*H. almeriensis* strain M8^T^ secretes a polymer with varying molecular weights (6.3 × 10^6^ Da and 1.5 × 10^4^ Da). The high-molecular-weight fraction is composed of Man, Glc, and Rha, while the low-molecular-weight fraction contains Man and Glc [85]. *H. anticariensis* FP35^T^, isolated from a saline wetland in southern Spain, yields EPS with a molecular weight of 50 kDa, primarily comprising Glc, Man, and Gal [86]. *H. ventosae* Al12^T^, isolated from a saline soil in Jaén, Spain, produces EPS associated with the cell surface and the surrounding medium, with a molecular mass of about 50 kDa and a composition of Glc, Man, and Gal [86].

Furthermore, *H. alkaliphila* strain 18bAG^T^, a halotolerant alkaliphilic bacterium from Campania, Italy, produces extracellular material rich in carbohydrates, particularly when in the stationary growth phase and cultivated in a medium containing maltose and yeast extract [87]. *Oceanobacillus oncorhynchi* subsp. *incaldanensis* strain 20AG^T^, an alkalitolerant halophile microorganism isolated from a sulfurous spring in Campania, excretes polysaccharide during the stationary growth phase when grown in a minimal medium containing trehalose [88].

*Alteromonas hispanica* F32^T^, isolated from a hypersaline habitat in Spain, is a moderate halophilic bacterium capable of producing an EPS with a molecular weight of 1.9 × 10^7^ Da, composed of glucose (GIc), mannose (Man), rhamnose (Rha), and xylose (Xyl) as monosaccharides, along with sulfate and phosphate groups [89]. *Bacillus licheniformis* strain B3-15, a halophilic and thermotolerant bacterium from Vulcano island, Italy, produces an EPS with a tetrasaccharide repeating unit primarily consisting of sugars in a mannopyranosidic configuration [90].

*Colwellia psychrerythraea* 34H, isolated from Arctic marine sediments, synthesizes extracellular and capsular polymers as cryoprotectants, with these polysaccharides containing amino sugars, uronic acids, and amino acidic decorations. Additionally, when incubated at 8 °C, it produces another polymer with a different monosaccharide composition unrelated to antifreeze activity [91]. *Salipiger nanhaiensis* ZH114T, a moderately halophilic, facultatively anaerobic bacterium from the South China Sea, is known for its EPS production [92]. *Salipiger mucosus* A3^T^, isolated from the Mediterranean seaboard, produces a heteropolysaccharide mainly during its exponential and stationary growth phases, characterized by a molecular mass of 250 kDa and composed of Glc, Man, Gal, and Fuc [93].

*Pantoea* sp. strain BM39, isolated from sediments in the Tyrrhenian Sea, is proficient at secreting exceptionally high levels of EPS, particularly when cultivated on glucose medium during exponential growth. This exopolymer consists primarily of glucose and has a substantial molecular mass [94]. *Chromohalobacter canadensis* 28, isolated from Pomorie salterns, is a moderately halophilic bacterium that produces an extracellular polymer substance, composed of an EPS fraction (14.3% *w*/*w*) and a protein fraction (72% *w*/*w*), with the EPS primarily comprising GlcN, Glc, Rha, and Xyl [95].

A novel EPS-producing bacterium, *Natronotalea sambharensis* sp. nov. strain AK103T, was successfully isolated from Sambhar Lake, Rajasthan, India. This strain exhibited significant EPS production (1.2 g/L) under optimal conditions, with the EPS composed of mannose, glucose, and glucuronic acid, demonstrating antioxidant properties and the ability to synthesize gold nanoparticles [96].

A novel EPS, named hsEPS, was extracted from the high-salt-fermented broth of *Halomonas saliphila* LCB169^T^. This EPS, primarily composed of mannose and glucose and with a molecular weight of 5.133 × 10^4^ g/mol, demonstrated remarkable water solubility, water-holding capacity, oil-holding capacity, foaming capacity, and emulsifying activity, suggesting its potential as a versatile ingredient in food, cosmetics, and detergents [97]. Another study focuses on the exploration of *Virgibacillus dokdonensis* VITP14’s EPS, with a substantial production of 17.3 g/L after 96 h of fermentation. The EPS, characterized by a porous web-like structure through elemental composition analysis and an identified monosaccharide composition (glucose, ribose, fructose, xylose), displays excellent water solubility, water-holding capacity, emulsifying properties, hemocompatibility, and cytocompatibility, making it a promising biomaterial for various applications in tissue engineering and beyond [98].

### 3.3. Chromohalobacter canadensis 28

*Chromohalobacter canadensis* 28, an obligate halophilic bacterium isolated from the saline environments of Pomorie salterns in Bulgaria, has attracted significant attention as a prolific producer of an extracellular polymer substance (EP) [95]. This EP has garnered interest for its potential applications in various industries, especially cosmetics. One of the key findings of this research was the identification of lactose, a sugar derived from the dairy industry, as the optimal carbon source and peptone as the optimal nitrogen source for EP production by *C. canadensis* 28. Maximum productivity was recorded at the end of a logarithmic phase, until the eighteenth hour. Through optimization of culture conditions and physicochemical parameters, researchers achieved a remarkable twofold increase in the biosynthesis of this unique EP. Notably, the bacterium exhibited its highest EP synthesis rates under unusually high NaCl concentrations, particularly at 150 g/L. This elevated salt concentration not only stimulated polymer production but also played a crucial role in preventing microbial contamination during the fermentation process [95]. 

Chemical analysis of the purified EP revealed a distinctive composition comprising two main fractions: an EPS fraction and a protein fraction, with polyglutamic acid (PGA) dominating the protein component. The EPS fraction, which accounted for 14.3% *w*/*w* of the EP, displayed an intriguing sugar composition. It primarily consisted of glucosamine, glucose, rhamnose, and xylose, setting it apart from previously reported EPS compositions. This novel EPS exhibited a wide range of valuable properties. It demonstrated high water solubility, an exceptional water-holding capacity, robust emulsifying activity, and remarkable foaming capacity. Furthermore, it displayed antioxidant activity and the unique ability to synthesize gold nanoparticles, adding to its potential versatility [95].

The next study also introduced an innovative approach to enhance EP synthesis by *C. canadensis* 28 using continuous cultures. This method proved highly effective, yielding EP levels similar to those achieved in batch cultures (2.4 g/L), but with the significant advantage of avoiding the challenges associated with discontinuous fermentation processes. The process parameters remained stable even after a ten-day run, showcasing the robustness of this approach [99].

Furthermore, the EP’s biocompatibility and its potential applications in the cosmetics industry were explored. In vitro experiments using human epidermal keratinocyte cells (HaCaT) and dermal fibroblast cells (PCS-201-012) demonstrated the positive effects of both crude EP and purified γ-PGA on cellular proliferation and the expression of genes related to collagen, hyaluronic acid, involucrin, and filaggrin [100]. These findings suggest that the EP has the potential to improve the skin barrier, stimulate collagen and hyaluronic acid production, and facilitate wound closure, making it an attractive candidate for cosmeceutical products.

In conclusion, these studies have shed light on the remarkable potential of *Chromohalobacter canadensis* 28 and its unique extracellular polymer substance for various applications, particularly in the cosmetics and skincare industries. Its high hydrophilicity, antioxidant properties, and the presence of glucosamine make it a valuable asset for skin health. The development of advanced cosmeceutical and skincare products incorporating this EP and purified γ-PGA appears promising, given their demonstrated biocompatibility and positive effects on skin-related cellular processes. This research opens doors to innovative uses of halophilic microorganisms in the production of natural, biocompatible compounds for cosmetic and biomedical applications.

As the research on *C. canadensis* 28 continues to unfold, the industrial and biotechnological potential of its EP beckons exploration into novel applications and future advancements. The unique characteristics of the EP, ranging from its sugar composition to its ability to synthesize gold nanoparticles, present an exciting array of possibilities for various industries. Industries such as food and pharmaceuticals may benefit from its water solubility, emulsifying activity, and foaming capacity, suggesting potential applications beyond cosmetics [101]. In the realm of biotechnology, the continuous culture approach has demonstrated not only effectiveness but also stability, showcasing the robustness of the method for sustained EP production. This opens avenues for streamlined and efficient large-scale production processes, reducing challenges associated with discontinuous fermentation. Looking forward, the EP’s biocompatibility and positive effects on cellular processes make it a compelling candidate for further exploration in biomedical applications [102]. The observed impact on cellular proliferation and gene expression related to essential skin components paves the way for innovative developments in wound healing and tissue regeneration. The unique combination of properties, including antioxidant activity, positions the EP as a multifaceted tool in the development of advanced materials and therapeutic interventions [103,104].

Furthermore, the research on *C. canadensis* 28 sets the stage for broader investigations into halophilic microorganisms and their untapped potential. Future studies may delve into the genomics and metabolic pathways of such microorganisms, unlocking new possibilities for genetic and metabolic engineering to tailor their properties for specific applications. In essence, the studies on *C. canadensis* 28 not only unravel the current applications of its EP but also beckon researchers and industries to envision a future where these unique polymers play a pivotal role in diverse fields. The dynamic interplay between scientific exploration and practical applications promises a wealth of opportunities for harnessing the capabilities of halophilic microorganisms in ways that were previously unimagined.

**Table 2:** Halophiles, carbon source, monosaccharide analysis, properties and activities, EPS production levels.

Halophiles (Specific Conditions)	Carbon Source	Monosaccharide Analysis	Properties and Activities	EPS Yield (g/L)	References
*Chromohalobacter canadensis* 28 (30 °C, pH 7.5, 150 g/L NaCl)	Lactose	Glucosamine/glucose/rhamnose/xylose/unknown sugar (36.7/32.3/25.4/1.7/3.9, by weight percentage)	Pseudoplastic rheological property; high swelling behavior; emulsifying and stabilizing activities; foaming ability	2.4	[95,99,100]
*Halobacillus.* sp. Strain EG1HP4QL (35 °C, pH 8.0)	Sucrose	Two polymers, a negatively charged and a neutral one (~3:1), in which mannose and glucose are the main neutral monosaccharide constituents	Emulsifying activity	5.9	[105]
*Halomonas smyrnensis* AAD6T (37 °C, pH 7.0, 137.2 g/L NaCl)	Sucrose	Fructose	Degradation temperature 253 °C; bioflocculating activity; anti-cytotoxicity; biocompatibility; antitumor activity after periodate oxidation	1.073—flasks and 1.844—fermenter	[79,80,81,106,107,108]
*Halomonas almeriensis* M8T (32 °C, pH 7.0, 75 g/L)	Glucose	EPS 1, mannose/glucose/rhamnose (72/27.5/0.5, by weight percentage) EPS 2, mannose/glucose (70/30, by weight percentage)	Emulsifying activity; heavy metal binding capacity; pseudoplastic rheological property	1.7	[85]
*Halomonas stenophila* B100 (32 °C, pH 7.2, 75 g/L total salts)	Glucose	Glucose/galactose/mannose (44.5/40.5/15.0, by weight percentage)	Antitumor activity after oversulfation	3.89	[76,77]
*Alteromonas hispanica* F32T (32 °C, pH 7.2, 75 g/L total salts)	Glucose	Mannose/glucose/xylose/rhamnose (62.75/18.15/12.25/6.85, by molar percentage)	Emulsifying activity; heavy metal binding capacity; pseudoplastic rheological property	1–1.5	[89]
*Halomonas eurihalina* F2-7 (32 °C, pH 7.2, 75 g/L total salts)	Glucose	Glucose/mannose/rhamnose (2.9/1.5/1, by relative ratio)	Emulsifying activity; pseudoplastic rheological property	1.6	[78,109]
*Halomonas ventosae* A112T (32 °C, pH 7.2, 75 g/L total salts)	Glucose	Glucose/mannose/galactose (1.75/4/1, by molar ratio), and small quantities of xylose, arabinose, and galacturonic acid	Emulsifying activity; heavy metal binding capacity; biofilm formation capacity; pseudoplastic rheological property	0.28	[86]
*Halomonas anticariensis* FP35T (32 °C, pH 7.2, 75 g/L salts)	Glucose	Glucose/mannose/galacturonic acid (1/3/2.5, by molar ratio)	Emulsifying activity; heavy metal binding capacity; biofilm formation capacity; pseudoplastic rheological property	0.29	[86]

## 4. Conclusions

In conclusion, this review highlights the remarkable potential of extremophiles, particularly those found in Bulgaria’s diverse extreme environments, as a valuable resource for the production of EPSs with exceptional properties. These extremophiles have thrived in some of the most challenging conditions on Earth, and their adaptive strategies have led to the biosynthesis of unique biomolecules, including EPSs.

The research conducted in Bulgaria, focusing on both thermophilic and halophilic microorganisms, has yielded promising candidates for EPS production. Notably, strains such as *Geobacillus tepidamans* V264, *Aeribacillus pallidus* 418, *Brevibacillus thermoruber* 423, *Brevibacillus thermoruber* 438, and *Chromohalobacter canadensis* 28 have emerged as potential EPS producers. Through optimization of cultivation conditions and rigorous physicochemical analyses, the synthesized biopolymers have exhibited excellent properties, including emulsifying capabilities and stability, opening up new avenues for their application in industries such as cosmetics and medicine.

EPSs from extremophiles offer several advantages over traditional plant polysaccharides, including faster production, controlled yield, and a diverse range of chemical structures. These characteristics make extremophile-derived EPSs highly attractive for various industrial sectors, from food production to biotechnology. The study emphasizes the importance of exploring extremophile biodiversity to isolate reliable EPS producers, unlocking the full potential of these microorganisms in the world of biopolymers and biotechnological innovations.

In summary, the manuscript underscores the significance of extremophiles as a valuable source of EPSs with unique properties, presenting opportunities for innovation and development in multiple industries. The exploration of Bulgaria’s rich extremophilic resources not only contributes to scientific knowledge but also paves the way for novel applications and advancements in the industrial and biotechnological sectors.
